# Supplementary figures and images for: Novel Babesia bovis exported proteins that modify properties of infected red blood cells
Source: PLoS Pathog. 2020 Oct 5;16(10):e1008917. doi: 10.1371/journal.ppat.1008917 (PMC7561165; doi:10.1371/journal.ppat.1008917)

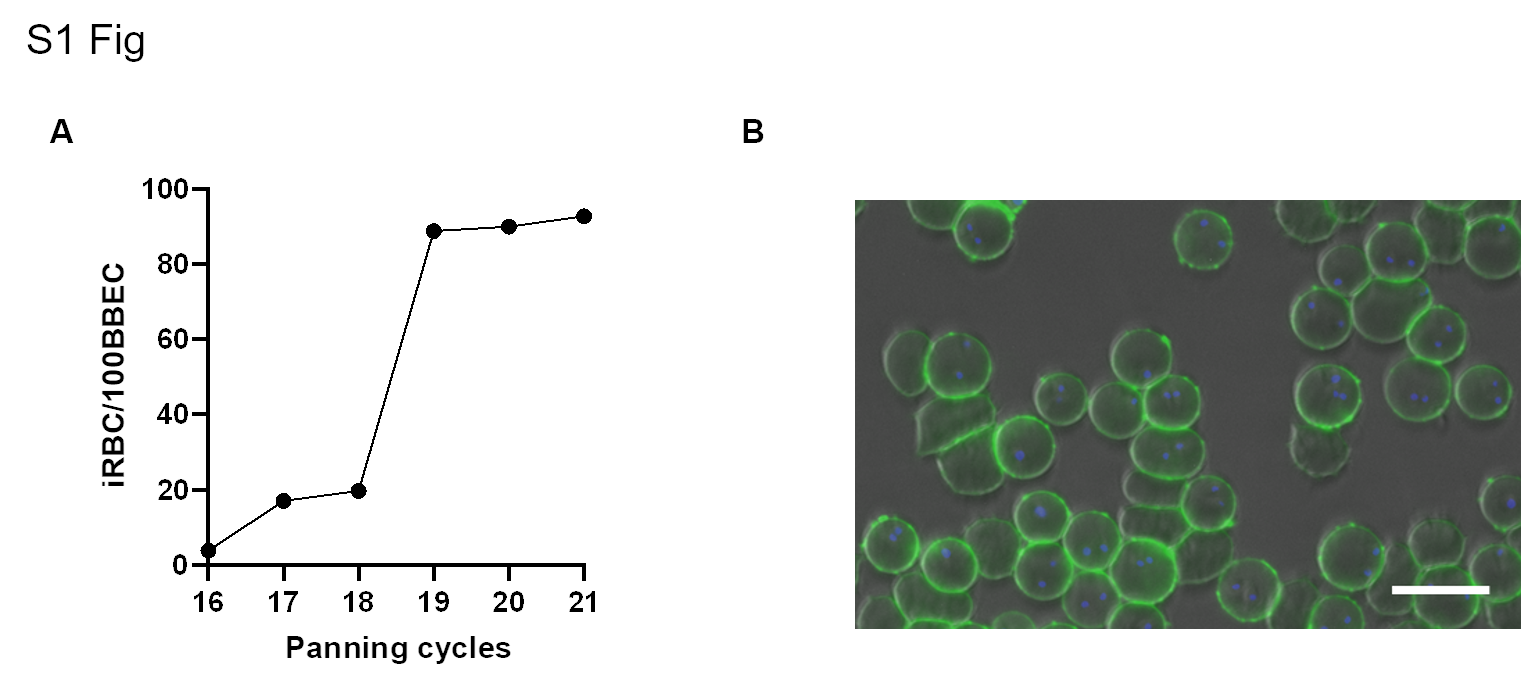

Supplement: S1 Fig — (A) Selection of cytoadherent B. bovis to BBECs. The number of bound iRBCs per 100 BBECs were counted. (B) Live fluorescence microscopy of biotinylated iRBCs reacted with streptavidin-conjugated Alexa Fluor 488 (green). The parasite nuclei were stained with Hoechst 33342 (Hoechst, blue). No released merozoites were seen. Scale bar = 10 μm. (TIF) [file ppat.1008917.s001.tif]

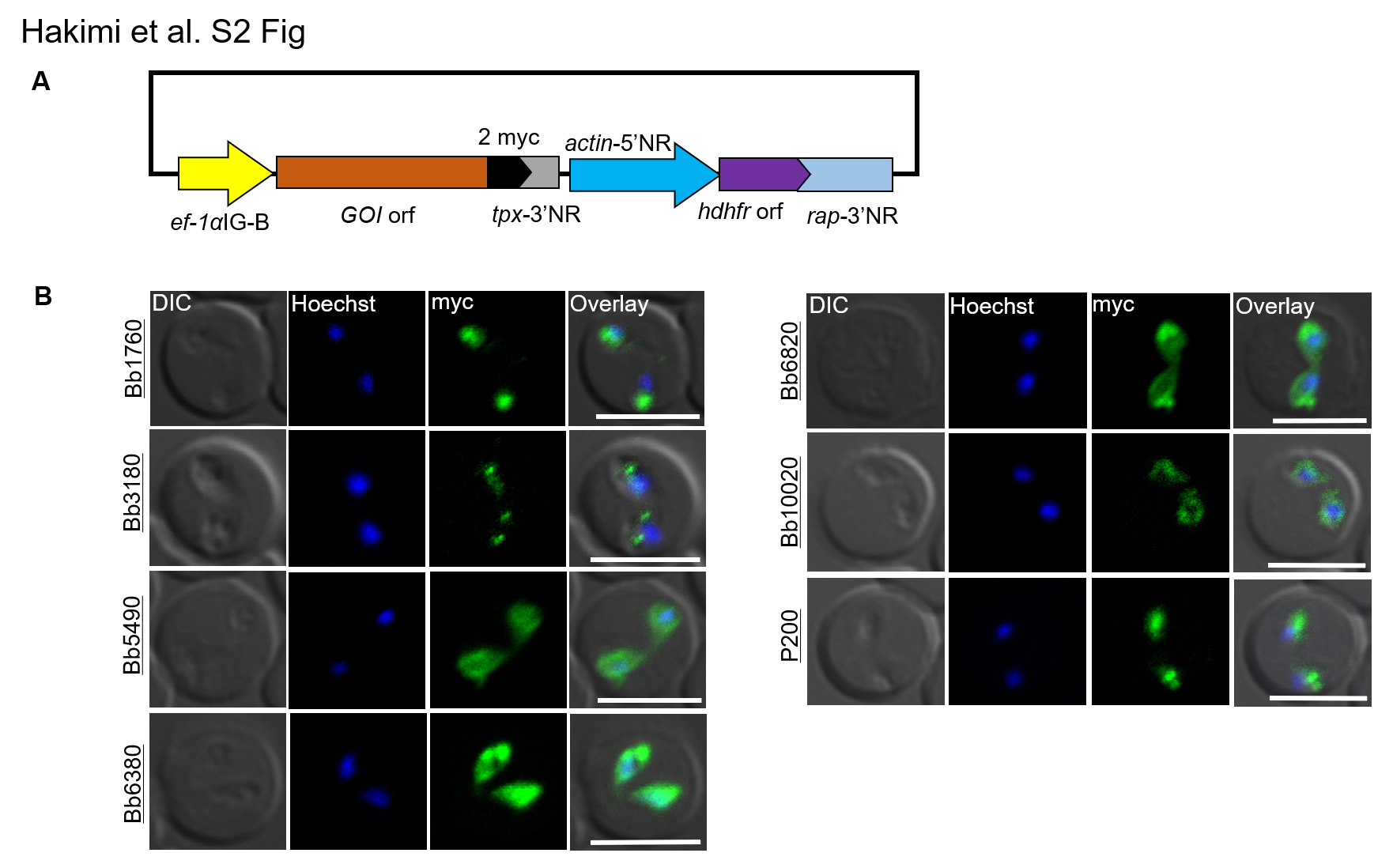

Supplement: S2 Fig — (A) Schematic of a plasmid expressing myc-tagged candidate proteins. ef-1αIG-B, elongation factor-1α intergenic region B; GOI orf, gene of interest ORF; tpx-3’NR, thioredoxine peroxidase-1 3’ noncoding region; hdhfr orf, human dihydrofolate reductase ORF; rap-3’NR, rhoptry associated protein 3’ noncoding region. (B) Indirect immunofluorescence antibody test of transgenic B. bovis expressing myc-tagged target proteins stained with anti-myc (green). The parasite nuclei were stained with Hoechst 33342 (Hoechst, blue). Scale bar = 5 μm. (TIF) [file ppat.1008917.s002.tif]

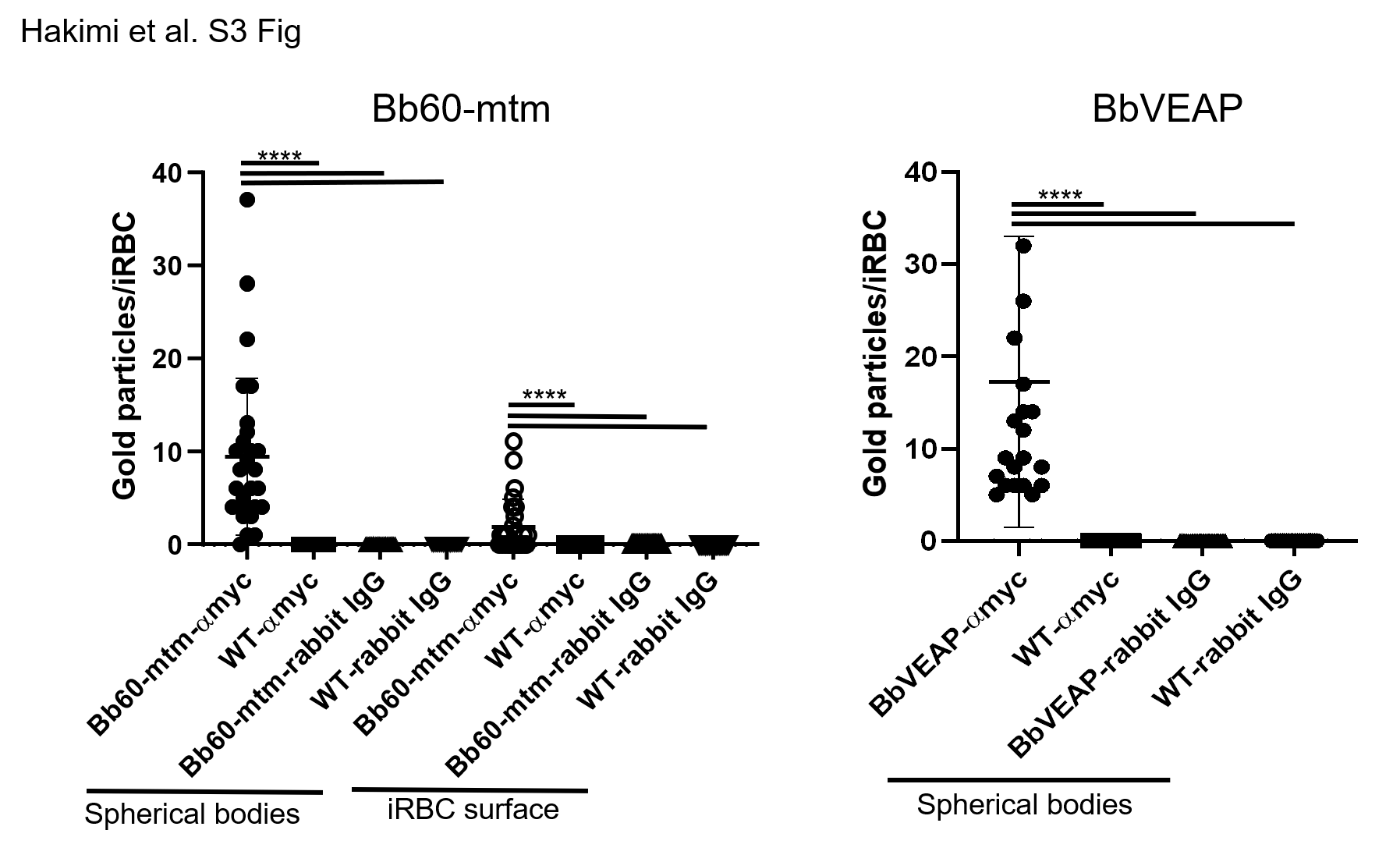

Supplement: S3 Fig — The number of gold particles were quantified in B. bovis with Bb60-mtm and BbVEAP tagged with myc epitopes. Gold particle numbers were counted in spherical bodies and iRBC surface only in parasites with clear spherical bodies in electron micrograph sections (****, P < 0.0001; determined by Mann-Whitney U test). (TIF) [file ppat.1008917.s003.tif]

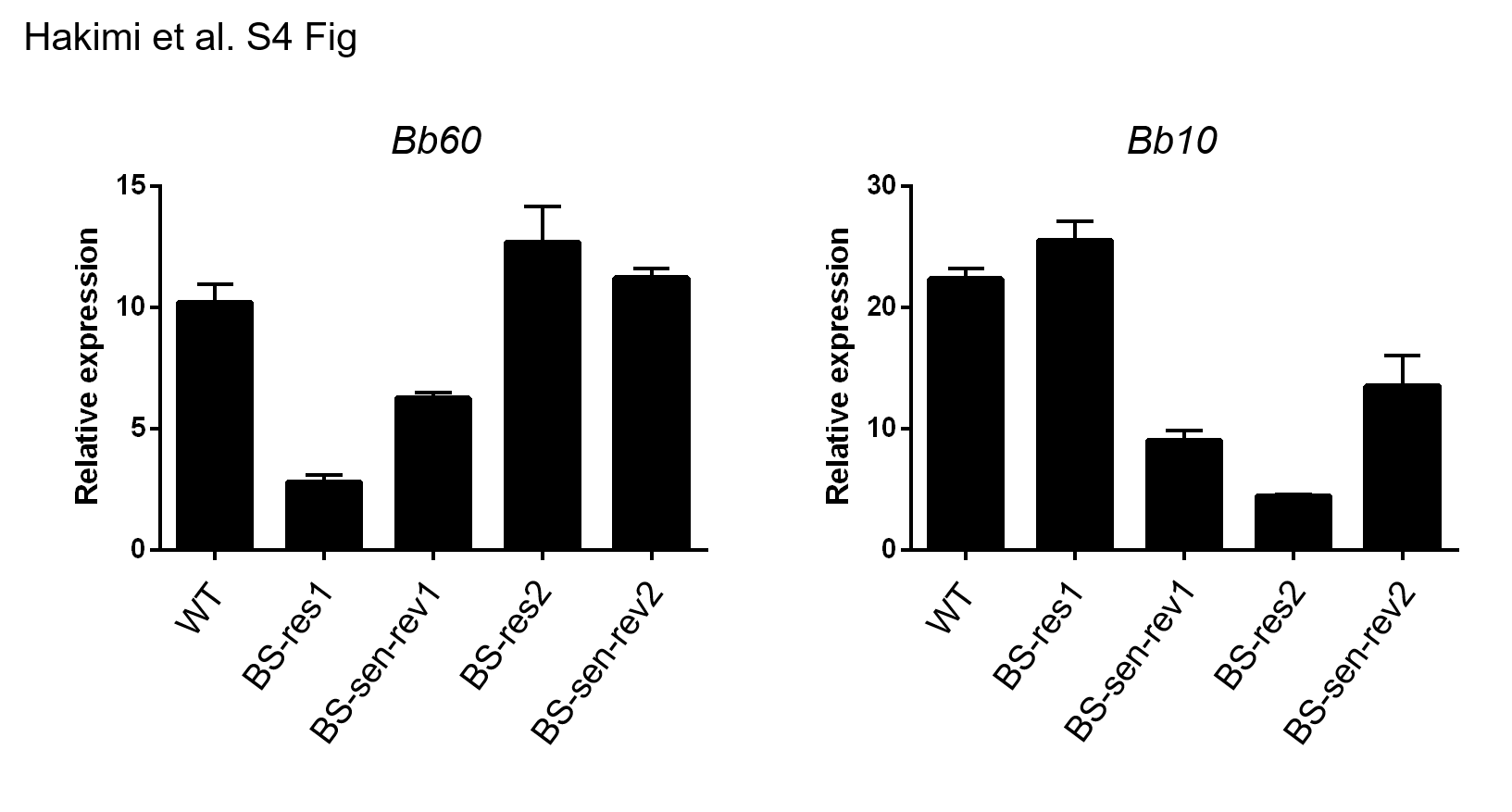

Supplement: S4 Fig — Relative transcript levels of Bb60 and Bb10 in WT, BS-resistant lines and BS-sensitive revertants. Transcript levels are normalized against methionyl-tRNA synthetase (Gene ID: BBOV_I001970). (TIF) [file ppat.1008917.s004.tif]

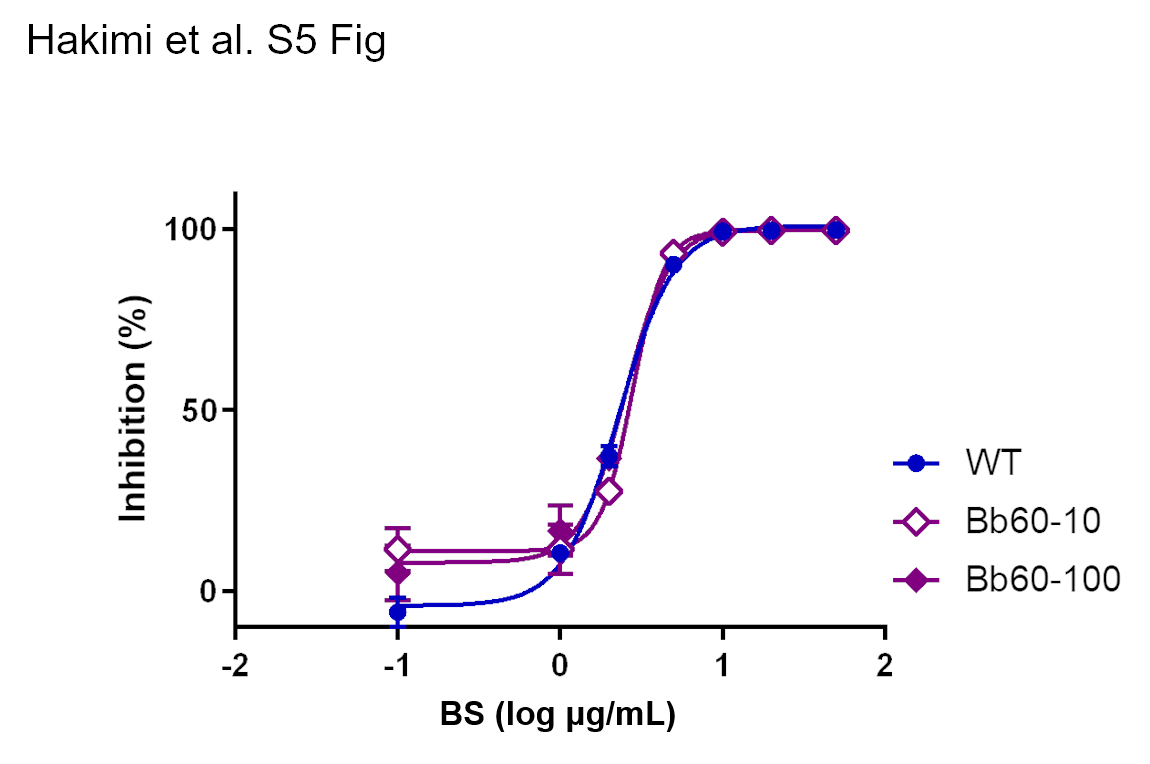

Supplement: S5 Fig — Growth inhibition curves of two Bb60-overexpressing parasite lines in the presence of different concentrations of BS (μg/mL). Bb60-mtm is episomally overexpressed in WT parasites under 10 or 100 nM WR99210 in Bb60-10 or Bb60-100 lines, respectively. All data are expressed as mean ± SEM. (TIF) [file ppat.1008917.s005.tif]

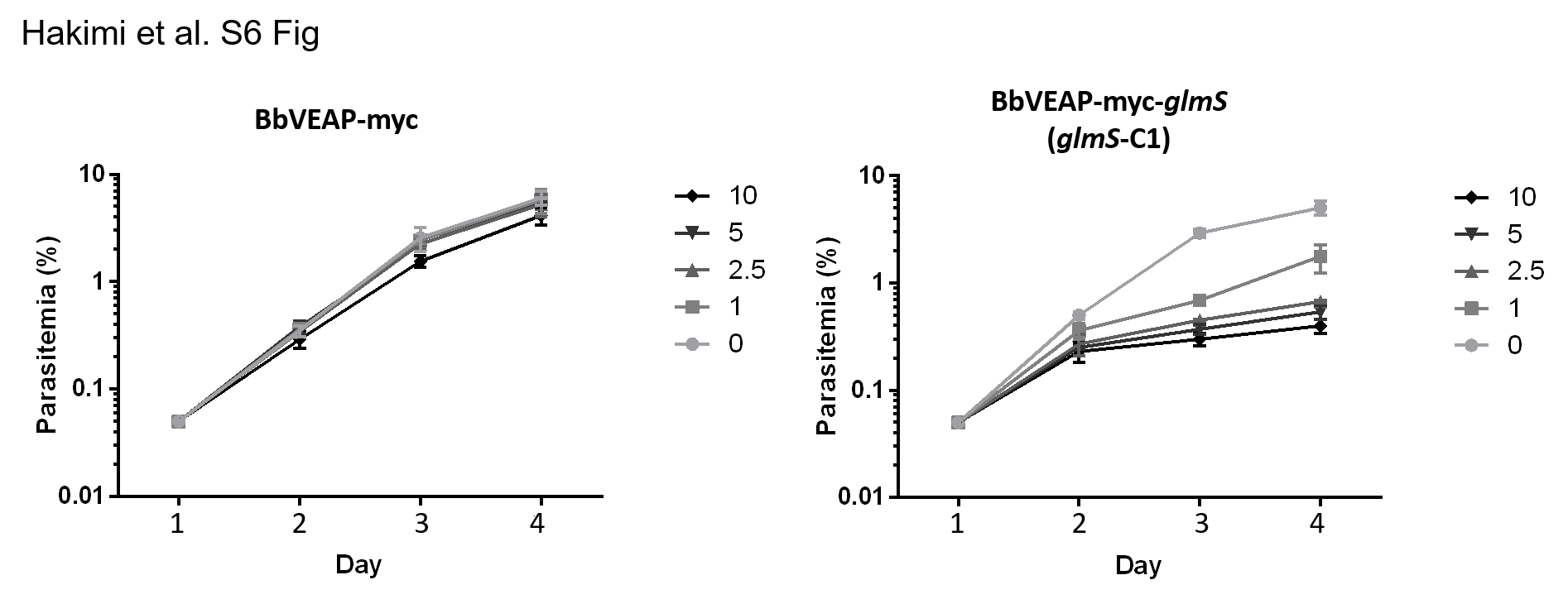

Supplement: S6 Fig — Growth of BbVEAP-myc-glmS and control parasites (BbVEAP-myc) in the absence or presence of 1, 2.5, 5, and 10 mM GlcN. Initial parasitemia was 0.05% and parasitemia was monitored for 3 days with daily culture medium replacement. The data are shown as mean ± S.D. from technical triplicates. (TIF) [file ppat.1008917.s006.tif]

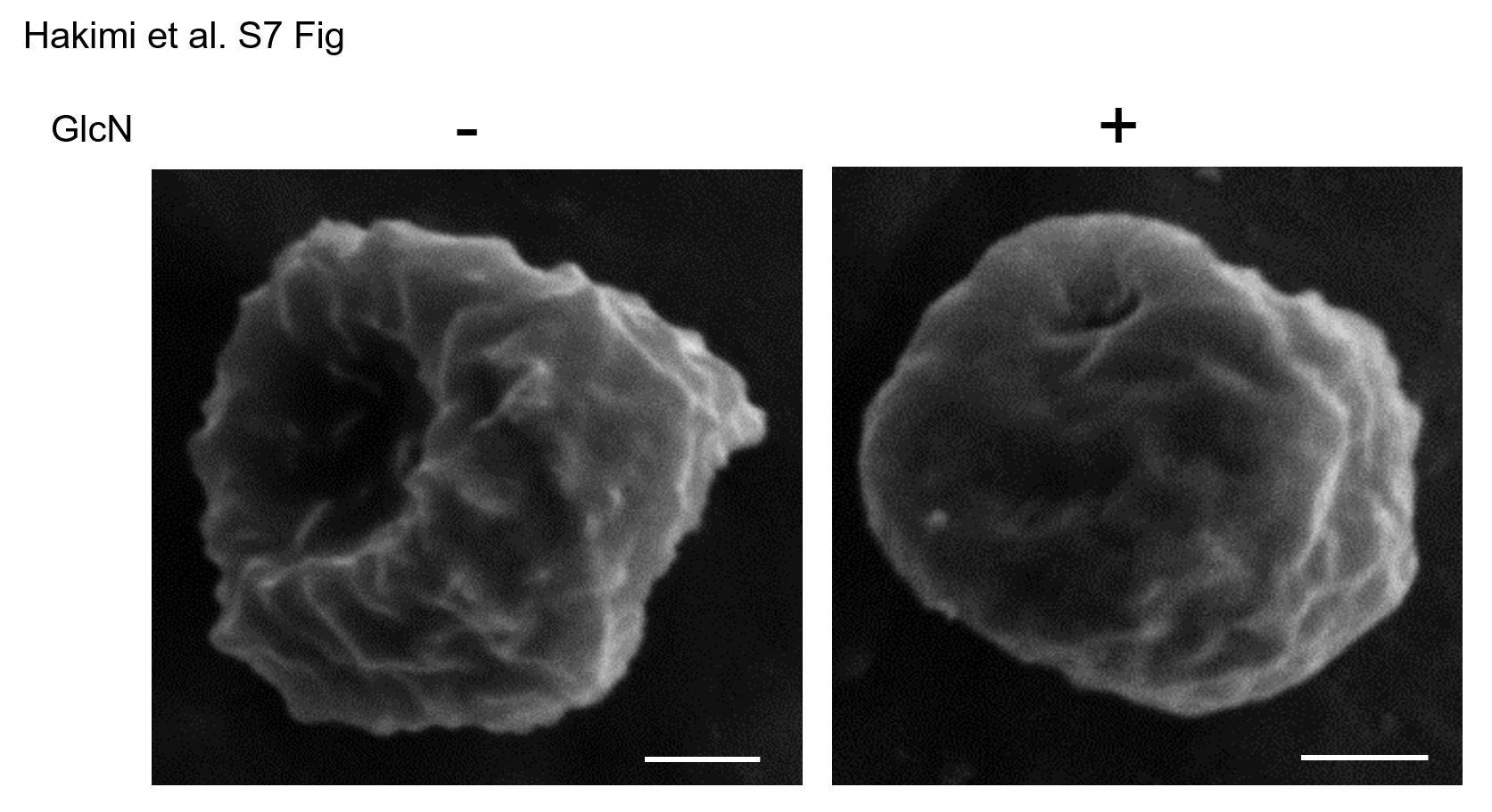

Supplement: S7 Fig — Scanning electron microscopy showing ridges on the surface of RBC infected with BbVEAP-myc-glmS parasites or a control parasite following 3 days exposure to GlcN. Scale bar = 1 μm. (TIF) [file ppat.1008917.s007.tif]

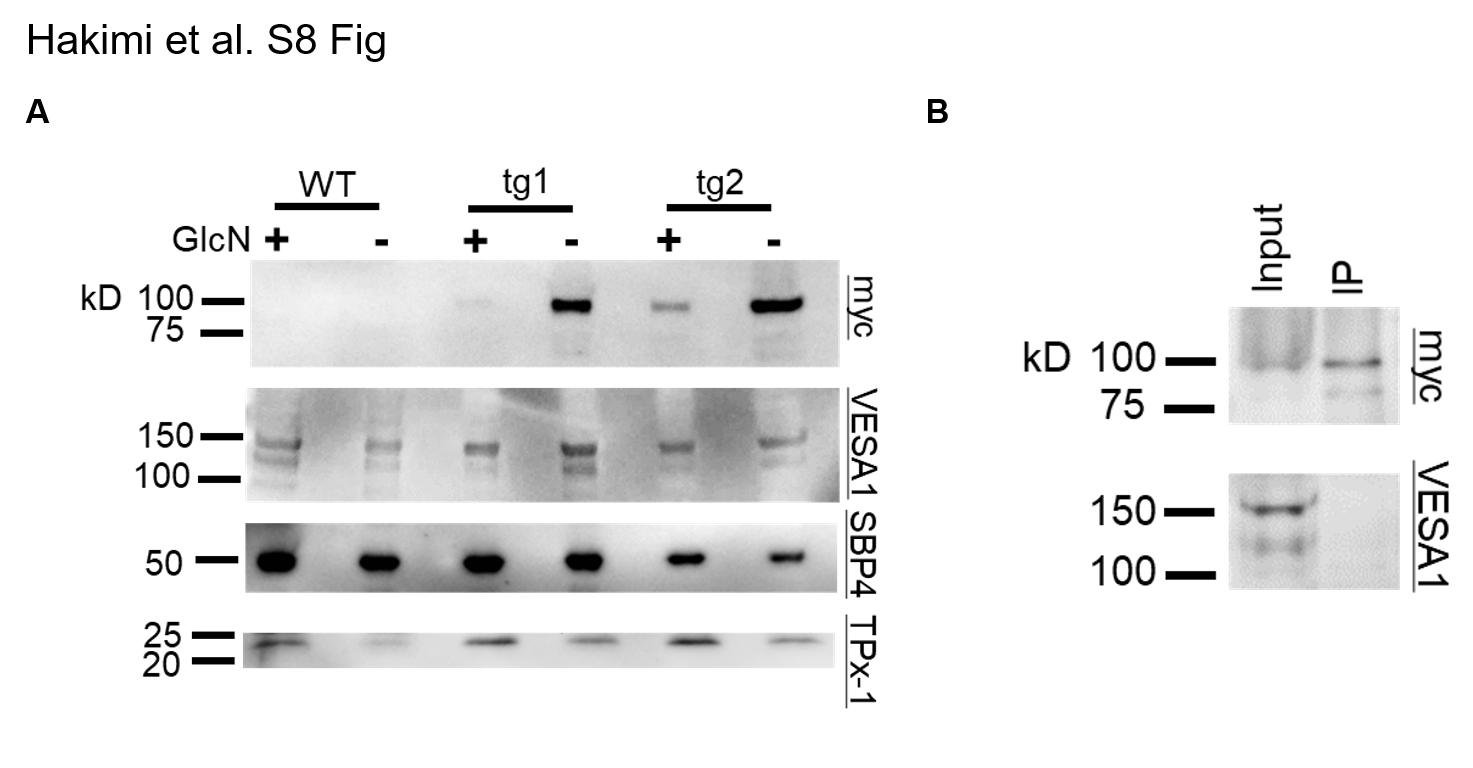

Supplement: S8 Fig — (A) Western blot analysis of two clones (tg1, tg2) of BbVEAP-myc-glmS and WT parasites in the presence or absence of GlcN. TPx-1 detected with anti-TPx-1 antibody was used as a loading control. (B) Immunoprecipitation with anti-myc for BbVEAP-myc parasite. (TIF) [file ppat.1008917.s008.tif]
