# Supplementary material for: Novel Babesia bovis exported proteins that modify properties of infected red blood cells
Source: PLoS Pathog. 2020 Oct 5;16(10):e1008917. doi: 10.1371/journal.ppat.1008917 (PMC7561165; doi:10.1371/journal.ppat.1008917)
Supplement: Attachment — Submitted filename: PPathogens.D-20-00279.review.docx [file ppat.1008917.s015.docx]

Points to be considered as they appear in the text:

1) Page 4, line 73: Invasion by tick injected sporozoites and merozoites?
2) Page 6, line 129 and Table 1: How many peptides were common to all 3 MS samples, as you only state that 80 were detected in 2 out of the 3?
3) Page 8, line 171: Could the quality (definition) of the all IFA images be improved?
4) Page 12, line 253: As Table S4 lists 102 genes downregulated and 96 genes upregulated how can the authors state that this clearly “links” Bb10 and Bb60 to BSD-resistance? How tenuous is the link?
5) Page 13, section Characterization of BbVEAP starting line 276: How do the authors reconcile their two apparently contradictory statements? On lines 277/278 they state that the failure to eliminate the gene for BbVEAP indicates its essentially for the parasite. Yet, on lines 284/285 they state an 80% reduction in basal BbVEAP expression did not affect parasite growth.
6) Lines 316-318: They state that 80-90% knockdown resulted in a significant decrease in growth rate accompanied by a significant increase of the ring stage…. Suggesting a defect in parasite growth and development. Can they clarify what they mean by growth versus development, as surely what they observe is rather just a block in development?
7) Page 15: As BbVEAP expression appears necessary for rings to progress to mature stages i.e. upon knockdown of BbVEAP rings accumulate, why did they count the number of ridges on mature stages? Do ring-infected RBC not express ridges? If ring stage-infected RBC don’t express ridges and knockdown leads to an accumulation of ring stage-infected RBC then this could explain why cyto-adhesion drops (Fig.6A).
8) Page 17, line 369: On could also write “Like Theileria the PVM ruptures within minutes of invasion by Babesia, since both are piroplasms.
9) Page 17, lines 385/386: BSD resistance was linked to the up and down regulated expression of 198 genes i.e. 196 genes in addition to Bb10 and Bb60. Some tempering required!
10) Page 18 line 393: It’s not clear that they have demonstrated BbVEAP is a virulence factor involved in ridge formation, as what they have demonstrated in that BbVEAP expression is necessary for rings to progress to mature stages.
11) Page 19, line 422: VARCSA is not variant it is in fact the only member of the Var gene family that doesn’t vary.
