# Supplementary material for: Novel Babesia bovis exported proteins that modify properties of infected red blood cells
Source: PLoS Pathog. 2020 Oct 5;16(10):e1008917. doi: 10.1371/journal.ppat.1008917 (PMC7561165; doi:10.1371/journal.ppat.1008917)
Supplement: Attachment — Submitted filename: Answers to reviewers_R1.docx [file ppat.1008917.s016.docx]

**ID: PATHOGENS-D-20-00279**

Article Title: Novel *Babesia bovis* exported proteins that modify properties of infected red blood cells

Authors: Hassan Hakimi, Thomas J Templeton, Miako Sakaguchi, Junya Yamagishi, Shinya Miyazaki, Kazuhide Yahata, Takayuki Uchihashi, Shin-ichiro Kawazu, Osamu Kaneko and Masahito Asada

We would like to express our gratitude to the editors and reviewers for their careful consideration of our manuscript. We have revised the manuscript according to the reviewers’ comments, described below and shown in red font in the revised manuscript.

**Reviewer #2**

**Firstly, the authors do not make it clear that the surface biotinylation approach resulted, like it did previously in other species, in leakiness of the RBC and/or stickiness of other, non-surface proteins, requiring drastical filtering of the 255 B. bovis hits, to come to a list of 38 proteins. This list still includes a number of known merozoite surface proteins, not only parasite proteins translocated to the RBC surface. Indeed, the authors show in S2 Fig, that 7 of the 10 uncharacterised proteins from that list, are localised to either apical organelles or the merozoite surface. Only 3 show the targeted export into the host cell the authors were looking for in their biotinylation experiment. Therefore, the authors should make it clear that the surface labelling did in fact label most abundant proteins, both within the RBC and probably also within the parasite and therefore the list of 38 resembles a list of proteins for which masspec evidence is available in blood stages and which have additionally one of the following criteria: signal sequence, TM domain or ER retention signal.**

Thank you for the comment. In *P. falciparum*, it is known that furosemide can prevent new permeability pathway activity of iRBCs (Staines et al., 2004; Sharling et al., 2007). No such inhibitors are known to block the putative transporters on the surface of *B. bovis* iRBCs. Thus, there is a chance of biotin entrance into iRBC and possibly parasite and biotinylation of proteins. Indeed, the mass spectrometry data detected several merozoite surface and apical proteins. In revision we explain this issue in the discussion as:

It is noted that our method resulted in the biotinylation and identification of merozoite surface and abundant proteins (S2B Fig), possibly due to biotin entry into or lysis of RBCs and parasites (line 393-396).

**Secondly, the flow of the manuscript is lacking. The authors could improve this by highlighting that once they generated the list of expressed proteins in B. bovis which have one of the 3 criteria stated above, that they focused on 2 of the genes from their list of 38 which showed export into the RBC.**

We modified the manuscript to connect the proteomics data with target proteins characterization, specifically we state:

Thus, we decided to further characterize three novel proteins that were exported to iRBC (line 186-187).

Of the three novel proteins that were exported into iRBC, Bb60-mtm and Bb11920-mtm are paralogous proteins with similar architectures of ten TM domains each and molecular weights of approximately 49 kDa (212-214).

**One of these is a member of the novel multigene family of potential transporters, which is a very interesting finding. The authors state that in B. bovis this list has 44 members but fail to include this list in the supplementary materials.**

In revision we have added a list of 44 mtm family members in the supplementary materials as S4 Table.

**In order to demonstrate that at least some of these MTMs are not only localized to the RBC membrane but also function as transporters the authors conduct RNA-seq analysis on BSD resistant B. bovis lines, identifying reduced expression of Bb60 and Bb10. This is comparable to studies in Plasmodium falciparum where the same has been described. Presumably Bb10 is a member of the MTM family which was not identified by the masspec approach? Also, presumably the authors tried to generate knockouts of Bb60 without detecting a growth or adhesion phenotype? Did the authors follow up on the function of Bb11920, but failed to find a phenotype and hence dropped the characterisation?**

Bb10 is a member of the mtm A-type (S4 Table) but was not identified in our LC-MS/MS; however, RNA-seq data showed that several mtms are expressed in BS sensitive or resistant parasites (S5 Table). Because mtm is a multigene family with 44 members, which could functionally complement each other, we think that conventional knock out strategy may not be feasible to study the function of these genes. Thus, we decided to make a blasticidin (BS)-resistant parasite line, assuming that BS uptake occurs through the putative transporter on the surface of iRBC. RNA-seq data showed that the expression of Bb11920 was not affected in BS-resistant parasites, thus we did not mention it in the manuscript.

**Following on from two MTM family members the authors then started to characterise VEAP, again without a clear link why the reader has to jump to this molecule next. The authors should clearly state that the chosen proteins for characterisation are the ones which they demonstrated earlier of being exported. To characterise the function of VEAP, the authors use very elegantly the GlmS system to inducibly down-regulate the transcript levels upon glucosamine addition. Unclear is however why even before GlcN addition the protein levels are reduced by 80% compared to wt. Could the authors please address this and speculate?**

We modified the manuscript to connect the sections as follows:

Our three attempts to disrupt the BbVEAP gene locus, the third protein in our list which exported into iRBC, using CRISPR/Cas9 system were unsuccessful, suggesting possible essentiality for the parasite (296-298).

We added an explanation on reduced expression of BbVEAP before adding GlcN as: The reduction of the BbVEAP protein expression without addition of GlcN could be due to a leakiness of the *glmS* system or endogenously produced GlcN by *B. bovis* as is reported for *Trypanosoma cruzi* [36], an observation for future verification (line 306-309).

**Reviewer #3:**

**While the identification of Bbmtm gene products as novel host cell membrane proteins is convincing and the comparative analysis of this family in Babesia and related parasites is important, the functional insight into these proteins is very preliminary. These findings should either be substantiated (overexpression of Bb60 or Bb10 in the blasticidine resistant lines would be an easy experiment to do, see major points) or the conclusions on the function of these proteins needs to be toned down or even removed.**

Thank you for this comment. We developed new transgenic BS-resistant lines overexpressing Bb60 and Bb10. Episomal overexpression of Bb60-mtm and Bb10-mtm in BS-resistant line 1 and 2 made these parasites more sensitive to BS (IC_50_ of 7.8 and 2.3 µg/mL for BS-res1-Bb60 and BS-res2-Bb10, respectively), supporting our hypothesis on their role in BS uptake (S5 Fig, Line 273-276).

**The data on VEAP is stronger but the influence on VESA1 is not very broadly documented and should also be substantiated.**

We added Western blot confirming that the expression of VESA1 is not affected by VEAP knockdown and only the localization of VESA1 is affected (S8A Fig, line 368-369).

**Part II – Major Issues: Key Experiments Required for Acceptance**

**Reviewer #3: Major points:**
**1. The data on a role of Bbmtm gene products in nutrient permeability of the host cell is not very conclusive. The authors raise parasites resistant to blasticidine and show that this is due to a reduced permeability of the membrane of infected erythrocytes. Transcriptomics with these parasites showed that in the two blasticidine resistant clones either Bb60 or Bb10 (both Bbmtm members) were downregulated - but among quite a large list of other genes, some of which were much more downregulated. Causality between downregulation of a Bbmtm and the phenotype is therefore not established. For instance VESA1 is downregulated much more in both cases, but likely is not the reason for the observed phenotype. The same is the case for many other downregulated genes. Bb10 and Bb60 are also not the most dramatically regulated genes.**

The expression of Bb10 and Bb60 were downregulated in BS-resistant parasites and partially recovered in the revertant BS-sensitive parasites. Additionally, we developed new transgenic BS-resistant lines overexpressing Bb60 and Bb10. Episomal overexpression of Bb60-mtm and Bb10-mtm in BS-resistant line 1 and 2 made these parasites more sensitive to BS (IC_50_ of 7.8 and 2.3 µg/mL for BS-res1-Bb60 and BS-res2-Bb10, respectively), supporting our hypothesis on their role in BS uptake (S5 Fig, Line 273-276).

**Figure S3 shows that Bb10 is expressed more in Res1 than in the corresponding reverted parasites and this difference seems to be in a similar fold level to the upregulation of Bb10 in the Reverted 2 vs Res2 line, indicating that a certain level of randomness is involved.**

Because several members of mtm are expressed in each parasite lines (S5 Table), the up and down regulation of one mtm may affect the expression of other members.

**To establish the role of the Bbmtm in the nutrient permeability of the infected erythrocyte more experiments are needed. Instead of overexpressing Bb60 in wild type parasites, overexpression of the downregulated Bb60 or Bb10 should be carried out in the R1 and R2 lines. Only if this reverts the phenotype, a function of these proteins in the channel activity can be postulated. This experiment is essential to draw the conclusion that these proteins are involved in this activity, otherwise these statements need to be removed from the paper (e.g. such as line 385...: ‘This resistance was linked to the expression of one mtm (Bb10 and Bb60 in each line) indicating that mtm is responsible for BSD uptake and may form a channel or transporter on the surface of iRBC...’).**

As suggested, we developed new transgenic BS-resistant lines overexpressing Bb60 and Bb10. Episomal overexpression of Bb60-mtm and Bb10-mtm in BS-resistant line 1 and 2 made these parasites more sensitive to BS (IC_50_ of 7.8 and 2.3 µg/mL for BS-res1-Bb60 and BS-res2-Bb10, respectively), supporting our hypothesis on their role in BS uptake (S5 Fig, Line 273-276).

- **If Bbmtm indeed turn out to play a role in the nutrient channel function of the host cell, I wonder why only very few Babesia species have an expanded family of these proteins if they have this function.**

Gene families that encode multi-transmembrane proteins are expanded in a lineage-specific pattern, but expansions are seen in multiple species of *Babesia*. Thereby, what is conserved is not orthology but the presence of lineage-specific expansion of genes encoding putative channels.

- **The fact that (line 364...): ‘VESA1, SmORFs, and Bbmtms are exported proteins and close association of their gene loci in the genome of B. bovis may suggest a common epigenetic control of expression.’ might also be used for a reversed argument and could explain why Bbmtms were downregulated, even though they may not have a function in this activity (if another neighbouring gene is involved in this activity and all other genes in the vicinity are epigenitically co-regulated).**

We checked the genes neighboring downregulated mtms confirming two genes neighboring Bb60, BBOV_III000050 (SmORF) and BBOV_III000070 (membrane protein, putative) were also downregulated with a log2 fold change of -1.8 and -1.3, respectively. However, for both genes the downregulation was less prominent than Bb60 (log2 fold of -2.5). In the case of Bb10, the neighboring genes were not affected. Taken together, we believe that the downregulation of mtm is caused by BS resistance and not co-regulation of neighboring genes.

**2. The functional data on BbVEAP is more direct and more broadly founded than that on Bbmtm. The conditional knock down of this protein convincingly shows a reduced presence of ridges and reduced cytoadherence. However, the validation of the knock down is only shown for the non-cytoadherent parasites. The full validation should also be shown for the cytoadherent parasites (this could for instance be provided as a supplementary figure). More information should also be given about this cytoadherent line (origin; characteristics; was it used before?). Furthermore the effect on VESA1 needs to be more thoroughly analysed. In Fig. 6b, in the GlcN+ parasites the VESA1 signal simply seems very faint. Is there a real difference in the location? Can the change in distribution (or expression levels?) of VESA1 be substantiated and quantified (IFA and Westernblot)? Is there a change in the distribution of VESA1 on the erythrocyte surface in EM?**

We added information regarding the origin of the cytoadherent parasites. We selected *B. bovis* for adhesion to bovine brain endothelial cells (BBECs) by panning uncloned parasites and the resulting cytoadherent parasite line was used for biotinylation (S1A Fig, line 122-124). We used a cloned parasite from the cytoadherent *B. bovis* line (S1A Fig) to generated additional transgenic parasite lines in which a myc-*glmS* sequence was inserted to the 3' end of the BbVEAP ORF the same as BbVEAP-myc-*glmS* line (line 360-363). We added Western blot data confirming knockdown of VEAP following addition of GlcN, while VESA1 and SBP4 expression in the cytoadherent parasites were unchanged (S8A Fig, line 365-366, 368-369).

**How do the authors explain that VEAP appears to be soluble in the host cell but was detected by the surface biotinylation? What does this mean for the other hits found in their mass spectrometry analysis? How can VEAP influence VESA1 and ridge formation in this location?**

VEAP may be pulled down with some biotinylated iRBC surface proteins that it interacts with or was biotinylated due to biotin entry into iRBC. We discussed this possibility in lines 393-396.

Although distribution of VESA1 in the iRBC was affected by BbVEAP knockdown, the mechanism behind of this observation is still unclear. Because we could not co-precipitate these two proteins by immunoprecipitation experiment (S8B Fig), they may not directly interact (436-440). The export and localization of ridge proteins may be linked to VEAP expression which needs to be validated in future studies.

**Part III – Minor Issues: Editorial and Data Presentation Modifications**


**Reviewer #2:**

**- Include number of unique peptides identified per protein in table S1**

We added the unique peptides per identified proteins in Table S1.

**- Line 126 states that a complete list of all identified proteins is given, it should read all identified B. bovis proteins as the host proteins are not listed.**

This sentence is corrected (line 129-131).

**- Include a list of all 44 B. bovis MTM genes**

We added S4 Table listing all 44 mtms.

**- It would be desirable to verify that other MTM proteins identified by masspec in this study also localize to the RBC membrane. Why choose Bb60 and Bb11920 out of the list of 7 mtm family proteins?**

We chose Bb60 and Bb11920 based on the higher quantitative values in MS (S1 Table). Since these two proteins are B-type mtm and Bb10 which is A-type mtm (S5A Fig), we assume that at least A-type and B-type mtms are exported and localized on the RBC membrane.

**Reviewer #3:**
- **The authors report that 32 of the proteins identified by the surface biotinylation possessed a PEXEL-like export motif (RxLx or RxxLx). Please clarify where in the protein these motifs were found. In malaria parasites these motifs are only functional if in a specific range downstream of a signal peptide but this is different in T. gondii. What were the criteria for calling of such a motifs in this study? I could not find the information in the M&M how this was done.**

The location of PEXEL-like motif (PLM) within the 32 candidate proteins are listed in S2 Table. We checked the existence of PLM (RXL or RXXL) based on the report from Pellé et al (2015). Their criteria was existence of PLM downstream of a signal peptide (SP) within the first 150 amino acids at N-terminus. Because some exported proteins in *B. bovis* like VESA1 do not have SP and possibly their transmembrane (TM) domain works as a signal for translocation to endoplasmic reticulum, we also included TM domain-containing proteins. Though, most proteins in the list have PLM based on these criteria, some proteins had a relaxed PLM not at N-terminal such as VESA1 and BbVEAP. Whether this PLM is functional and cleaved prior the export needs further experimentation.

**- Twelve proteins, including two positive controls, were selected for validation (line 168... and Figure S2). Can the authors explain in the text how exactly these proteins were selected?**

We modified the manuscript to clarify this point as:

We selected 10 proteins from the candidate list based on the three criteria for secretion and their abundance in the MS analysis (S3 Table) and further evaluated with two positive control proteins, SBP3 and a variant of SmORF (line 170-172).

**- Please provide statistics for the gold particle counts shown in Fig. 2c (how cells were selected; number of cells inspected; location of gold etc)**

We used two controls for our IEM, one myc-tagged parasites incubated with rabbit IgG and another WT parasites incubated with anti-myc antibody or rabbit IgG. We counted the gold particles only in the parasites that showed clear spherical bodies in the sectioning of IEM (S3 Fig).

**- Why do Bbmtm A and B cluster so far apart in 3c? Does this mean they are two independent families? What is the level of sequence homology?**

Among the 44 mtms, 10 members are clustered as B-type and 16 are grouped as A-type (S4 Table). B-type mtms have amino sequence identity values of 30-70% while A-type mtms are 65-76% identical. Bb10 belong to A-type cluster and Bb60 is B-type mtm with 16% amino acid sequence identity. Although the sequence of mtms are relatively diversified, structural and functional evidence suggest that A and B clusters belong to the same family.

**- Figure 5a PCR gel. I assume these are cloned parasites. If not, it should be shown that there are no parasites without the correct genomic insertion using a PCR to detect the unmodified locus (to exclude the presence of parasites with incorrect insertions). What is tg1-1 and tg1-2 etc in this figure part?**

We performed two independent transfections and cloned the transgenic parasites from each transfection. The PCR results are from two clones from each transfection. We added this information in the Methods section (line 558-559). We renamed the transgenic parasites to unify with the Western blot. *glmS*-C1 and *glmS*-C2 indicate transgenic lines independently generated and following "-1" and "-2" indicate 2 independent clones (line 326-327).

**- there is a ＇data not shown＇ in the discussion, please either provide the data or remove**

We included immunoprecipitation results in the supplementary materials and modified the manuscript accordingly (S8B Fig, line 436-440, line 603-614).

**- indicate at least fixative used for the IFA procedure (line 557 ＇air-dried and fixed as described [62]＇.), so the reader does not need to consult other publicatons to know the type of IFA that was used.**

We modified the sentence as: IFAT was done on thin blood smears from cultured parasites that had been air-dried and fixed in a 1:1 acetone:methanol mixture at −20°C for 5 min (line 617-618).

**- please provide the source of rabbit anti-SBP4 antibody (or provide validation if newly generated)**

We added the reference of anti-SBP4 used in this study (line 597).

**- is this a commercial rabbit anti-VESA1α antibody by MBL? If it is a custom made antibody, again validation of these antibodies needs to be provided**

It is a custom-made antibody and was validated using IFAT and WB with expected band size and expression pattern (S8 Fig and Fig 6B).

**- How were the CRISPR lines obtained? Were parasites cloned? If yes, how?**

All transgenic parasites were cloned by limiting dilution. We added this information in the Methods section (line 558-559).

**- exact information how often experiments were done and if technical or biological replicas are shown is often missing. Just as an example, the legend of figure 6 A says that all data are ＇triplicate assays＇. Are these independent experiments or technical replicas?**

We modified the text and clarified whether we have done 3 independent experiments or 3 technical replicates with the interval times between experiments (line 158-160, 283, 285-286, 331-333, 337-339).

**- please remove the statement in line 390-392: if this is true, this manuscript at least does not show it.**

In revision we removed this statement.

**Points to be considered as they appear in the text:**

**Page 4, line 73: Invasion by tick injected sporozoites and merozoites?**

This sentence was modified (line 73).

**Page 6, line 129 and Table 1: How many peptides were common to all 3 MS samples, as you only state that 80 were detected in 2 out of the 3?**

There were 35 proteins shared among three MS (Fig 1B).

**Page 8, line 171: Could the quality (definition) of the all IFA images be improved?**

We provided the original quality of IFA images and we hope these images are satisfactory.

**Page 12, line 253: As Table S4 lists 102 genes downregulated and 96 genes upregulated how can the authors state that this clearly “links” Bb10 and Bb60 to BSD-resistance? How tenuous is the link?**

Downregulation of mtm was seen in two independently developed BS-resistant lines. The expression of mtm were partially recovered in revertant parasites. Additionally, we developed new transgenic BS-resistant lines overexpressing Bb60 and Bb10. Episomal overexpression of Bb60-mtm and Bb10-mtm in BS-resistant line 1 and 2 made these parasites more sensitive to BS (IC_50_ of 7.8 and 2.3 µg/mL for BS-res1-Bb60 and BS-res2-Bb10, respectively), supporting our hypothesis on their role in BS uptake (S5 Fig, Line 273-276).

**Page 13, section Characterization of BbVEAP starting line 276: How do the authors reconcile their two apparently contradictory statements? On lines 277/278 they state that the failure to eliminate the gene for BbVEAP indicates its essentially for the parasite. Yet, on lines 284/285 they state an 80% reduction in basal BbVEAP expression did not affect parasite growth.**

We believe that 20% of VEAP is enough for parasite growth in normal *in vitro* culture condition. Whether this reduction affects the growth of the parasite *in vivo* should be validated in the future.

**Lines 316-318: They state that 80-90% knockdown resulted in a significant decrease in growth rate accompanied by a significant increase of the ring stage…. Suggesting a defect in parasite growth and development. Can they clarify what they mean by growth versus development, as surely what they observe is rather just a block in development?**

We corrected this sentence in the manuscript (line 316).

**Page 15: As BbVEAP expression appears necessary for rings to progress to mature stages i.e. upon knockdown of BbVEAP rings accumulate, why did they count the number of ridges on mature stages? Do ring-infected RBC not express ridges? If ring stage-infected RBC don’t express ridges and knockdown leads to an accumulation of ring stage-infected RBC then this could explain why cyto-adhesion drops (Fig.6A).**

It was shown that the number of ridges correlates with the parasite maturity (Hutchings et al., 2007); thus, to exclude the parasite stage on the number of ridges, we counted only the mature stage parasites. On the other hand, iRBC containing ring or mature stage of parasites are able to bind to endothelial cells (O’Connor et al., 1999), thus, we believe that abrogation of cytoadhesion is not due to the accumulation of ring stage iRBCs but results from mislocalization of VESA1 or other unknown ligands.

**Page 17, line 369: On could also write “Like Theileria the PVM ruptures within minutes of invasion by Babesia, since both are piroplasms.**

We modified this sentence in the manuscript (line 405-408).

**Page 17, lines 385/386: BSD resistance was linked to the up and down regulated expression of 198 genes i.e. 196 genes in addition to Bb10 and Bb60. Some tempering required!**

This sentence was modified as: We found only either *Bb60* or *Bb10* was downregulated in each of two BS-resistant lines among all *mtm* gene family members and revertants recovered the transcription level of respective *mtm*, indicating a link between expression of these *mtms* and BS resistance. These observation support the idea that Bbmtm acts as a channel or transporter and if this is the case, Bb60-mtm or Bb10-mtm is able to transport BS across the RBC membrane. Although *Bb60* and *Bb10* were downregulated in BS-resistant lines, the expression of other *mtms* were unchanged in comparison to control parasites, suggesting stable epigenetic regulation, or a substrate specificity for each *mtm*. Transcripts of several other genes were also reduced in the BS-resistant parasite lines, which might contribute to this resistance, and needs future evaluation (line 423-433).

**Page 18 line 393: It’s not clear that they have demonstrated BbVEAP is a virulence factor involved in ridge formation, as what they have demonstrated in that BbVEAP expression is necessary for rings to progress to mature stages.**

We have shown that knockdown of VEAP resulted in reduced RBC surface ridge numbers, mis-localized VESA1, and abrogated cytoadhesion to endothelial cells. Sequestration of iRBCs in brain is the main reason of fatal cerebral babesiosis which is caused by cytoadhesion of iRBCs to endothelial cells of brain microvasculature. Thus, we believe VEAP is a novel virulence factor for *B. bovis*.

**Page 19, line 422: VARCSA is not variant it is in fact the only member of the Var gene family that doesn’t vary.**

We modified this sentence in the manuscript as: PfEMP1-VAR2CSA which is expressed on the surface of *P. falciparum* iRBCs and is responsible for pathogenesis of pregnancy-associated malaria (line 463-464).

We hope that this revised manuscript be reconsidered and accepted for publication in PLOS pathogens.

Sincerely yours,

Hassan Hakimi

Masahito Asada
