# Supplementary material for: Novel Babesia bovis exported proteins that modify properties of infected red blood cells
Source: PLoS Pathog. 2020 Oct 5;16(10):e1008917. doi: 10.1371/journal.ppat.1008917 (PMC7561165; doi:10.1371/journal.ppat.1008917)
Supplement: Attachment — Submitted filename: Answers to reviewers_R1.docx [file ppat.1008917.s017.docx]

**ID: PPATHOGENS-D-20-00279R1**

Article Title: Novel *Babesia bovis* exported proteins that modify properties of infected red blood cells

Authors: Hassan Hakimi, Thomas J Templeton, Miako Sakaguchi, Junya Yamagishi, Shinya Miyazaki, Kazuhide Yahata, Takayuki Uchihashi, Shin-ichiro Kawazu, Osamu Kaneko and Masahito Asada

We would like to express our gratitude to the editors and reviewers for their careful consideration of our manuscript. We have revised the manuscript according to the reviewers’ comments, described below and shown in red font in the revised manuscript.

**Please note that Reviewer 3 has identified a few remaining points in need of correction before the manuscript could be accepted. Of particular importance are two points:**

**(i) Please try either to combine it into one of the main figures, or make it a main figure.**

We combined a part of S5 Fig with Fig 4 and they are now shown as panels E and F in the new Fig 4. Related descriptions in the main text are modified accordingly.

**(ii) Please respond to the comment regarding correction of labeling in S8 Figure to avoid any confusion.**

We corrected the order of the presence and absence not only in the S8 Fig legend but also the Fig 5 and 6 legends.

**Upon inspection, I observed two issues in S5 Figure: (i) the x-axes are labeled in “log ug/mL”, whereas in the figure legend the concentrations are provided in nM. Please make the units of concentration consistent throughout the manuscript (either system, but preferably in molarity).**

Blasticidin S (BS) is used in µg/mL concentrations to perform the growth assay. The plasmids were maintained episomally in transgenic parasites using WR99210 in nM concentrations. The Figure legend was modified to avoid confusion.

**(ii) In the lower panel of Figure S5B there are 4 curves but only 3 identified samples; please correct this. In addition, Figure S1B shows evidence of significant pixel shift between the DIC and fluorescent images. Please realign the superimposed images for correct alignment.**

We corrected S5 Fig and replaced S1B Fig with a better quality image.

***Part III – Minor Issues: Editorial and Data Presentation Modifications* Please use this section for editorial suggestions as well as relatively minor modifications of existing data that would enhance clarity.**

**Reviewer #2: (No Response)**

**Reviewer #3: The revised manuscript satisfactorily deals with the reviewer concerns. It contains new data that increases the support for the conclusions of the paper, including basic data on the adherent lines and overexpression data of Bb10 and Bb60 in the BS-resistant line.**

**I have only a few minor points left that I recommend to consider:**

**- The data in Fig. S5 is important (without it the evidence for Bb10 and**

**Bb60 in BSD transport is rather weak). Could it also be part of a main figure?**

We combined a part of S5 Fig with Fig 4 and they are now shown as panels E and F in new Fig 4. Related descriptions in the main text are modified accordingly.

**- + and - GlcN is mixed up in Fig S8**

We corrected the order of the presence and absence not only in the S8 Fig legend but the Fig 5 and 6 legends, too.

**- one further explanation for what the authors call 'leakiness' of the glmS line could be that insertion of the glmS sequence in the 3'-region affected the stability or translation of the target mRNA independent of its interaction with GlcN.**

As suggested, we modified the manuscript as:

The reduction of the BbVEAP protein expression without addition of GlcN could be due to a leakiness of the *glmS* system, decrease of mRNA stability or translation due to *glmS* sequence at its 3’ end, or endogenously produced GlcN by *B. bovis* as is reported for *Trypanosoma cruzi* [36], an observation for future verification (line 315-319).

**- Please get the manuscript checked by a native speaker: Some examples:**

**line 174: Two proteins showed signals inside parasite... add 'the'**

**before parasite**

**line 185: Remaining seven candidates did not show signals... add 'the'**

**at start of sentence**

**line 363: inserted to the 3' end of the BbVEAP ORF the same as BbVEAP-myc-glmS line. 'the same way as'?**

**line 366: with GlcN treatment by Western blot analysis (S8A Fig). 'as determined by Western blot analysis'?**

**line 426: These observation... observation should be plural line 429 the expression of other mtms were unchanged... 'was unchanged'**

Note from the second author, Tom Templeton: Sorry, my fault, I think that it is a well-written manuscript, but my mind's eye glossed over the errors. In revision the above errors are corrected and I have read it through again and remedied further mistakes.

We hope that this revised manuscript be reconsidered and accepted for publication in PLOS pathogens.

Sincerely yours,

Hassan Hakimi

Masahito Asada
